# Evaluation of Lymph Node Metastasis Among Adults With Gastric Adenocarcinoma Managed With Total Gastrectomy

**DOI:** 10.1001/jamanetworkopen.2020.35810

**Published:** 2021-02-10

**Authors:** Harbi Khalayleh, Young-Woo Kim, Hong Man Yoon, Keun Won Ryu, Myeong-Cherl Kook

**Affiliations:** 1 Center for Gastric Cancer, National Cancer Center, Goyang, Korea; 2 National Cancer Center Graduate School of Cancer Science and Policy, Goyang, Korea

## Abstract

**Importance:**

It is unclear whether proximal gastrectomy (PG) can replace total gastrectomy (TG), even in cases of advanced gastric carcinoma.

**Objectives:**

To evaluate the oncologic safety of PG based on the lymph node (LN) metastasis rate and develop a selection diagram for PG eligibility.

**Design, Setting, and Participants:**

In this cohort study, a retrospective analysis of a prospective database of gastric carcinoma surgery was performed including procedures that took place between December 1, 2000, and December 31, 2015, in the National Cancer Center, Korea, a high-volume carcinoma center with a structured database and accurate long-term follow-up. Among 9952 patients who underwent surgery for gastric carcinoma, 2347 underwent TG. Six-hundred fifty-five (564 in a second statistical analysis) had gastric carcinoma in the upper third of the stomach. The inclusion criteria were age 18 to 85 years, histologically proven adenocarcinoma (any size or differentiation) located in the upper third of the stomach, curative R0 TG performed, and postoperative follow-up for at least 3 years. Exclusion criteria included Borrmann type 4 carcinoma, T4 category, use of neoadjuvant chemotherapy, and a history of other carcinomas. Data analysis was performed from December 1, 2019, to May 30, 2020.

**Exposures:**

Total gastrectomy and LN dissection.

**Main Outcomes and Measures:**

The primary end point was the rate of LN metastasis at LN stations 4d, 5, and 6, which are usually not dissected during PG.

**Results:**

Among the 655 study patients, the mean (SD) age was 57.7 (11.9) years, and 462 (70.5%) were men. Only those with poorly differentiated cT3 category carcinomas had an increased incidence of LN metastasis at stations 4d (2 of 32 [6.3%]) and 11d (T3N0: 2 of 22 [9.1%], T3N1: 3 of 27 [11.1%]), independent of tumor size. For cT1-T3N0/1M0 category carcinomas, the incidence of station 5 LN metastasis was 0, irrespective of tumor size and differentiation. The LN metastasis rate at stations 4d and 6 for cT1-T3N0/1M0 differentiated tumors was also 0. Tumor size greater than or equal to 4.1 cm was associated with significantly increased LN metastasis compared with tumors less than 4.1 cm (40.0% vs 20.4%, *P* = .001).

**Conclusions and Relevance:**

The findings of this study suggest that PG can be safely performed for cT1-T2N0/1M0 tumors less than 4.1 cm in diameter that are located in the upper third of the stomach. The cT3N0/1M0-differentiated tumors less than 4.1 cm may also be eligible for PG, whereas poorly differentiated cT3 tumors and any cT4 or cN2/3 diseases require TG.

## Key Points

**Question:**

Under what clinical conditions is proximal gastrectomy associated with low risk of metastasis to regional lymph nodes?

**Findings:**

In this cohort study, including 655 patients who underwent total gastrectomy for gastric carcinoma in the upper third of the stomach, the rate of lymph node metastasis at station 5 was 0 for cT1-T3N0/1M0 regardless of tumor size and differentiation; rates for stations 4d and 6 for category cT1-T3N0/1M0 differentiated tumors were also 0. Tumor size of 4.1 cm or greater was associated with significantly increased lymph node metastasis compared with smaller tumors.

**Meaning:**

These findings suggest that proximal gastrectomy can be performed for category cT1-T2N0/1M0 tumors less than 4.1 cm with any histologic differentiation and for T3 category differentiated tumors less than 4.1 cm.

## Teaser

This cohort study evaluates the risk of lymph node metastasis of gastric carcinoma by category and size of the tumor and develops a selection diagram for use in determining whether proximal gastrectomy could be indicated.

## Introduction

The incidence of upper third gastric carcinoma is increasing.^1,2^ Total gastrectomy (TG) is recommended for upper third gastric carcinoma.^3,4^ Proximal gastrectomy (PG) is an alternative treatment of early gastric carcinoma and can achieve the same oncologic results as TG.^5,6,7,8,9,10^ Moreover, PG outperforms TG by various measures.^6,7,8,9,10,11,12,13,14,15,16^ A more anastomotic technique for PG has been developed, leading to an acceptable rate of anastomotic-related complications, thus further increasing the popularity of PG.^8,11^

The Japanese Gastric Cancer Association guideline identifies PG as an alternative for upper third, node-negative T1 tumors less than 4.0 cm in which half of the stomach can be preserved.^3^ However, LN metastases are rare at stations 4d, 5, and 6 for early gastric carcinoma, as well as for advanced T2 and T3 tumors.^17,18,19,20,21,22,23^ Yura et al^17^ reported a low metastatic rate for pathologic T2 and T3 tumors at stations 4d and 12a, and 0 for tumors at stations 5 and 6. Haruta et al^18^ reported that PG (excluding station 3b) could be safe and indicated in at least T2 tumors less than or equal to 4.0 cm and localized in the upper third of the stomach.

Therefore, classic guidelines should be reevaluated to address when PG can be performed safely. In this evaluation, the indication for PG was based on the clinical stage—not the pathologic stage. The study aim was to determine the risk of LN metastasis in each nodal station according to the clinicopathologic factors and use this risk to establish an indication for PG.

## Methods

This cohort study was a retrospective analysis of a prospective database of gastric carcinoma surgery performed between December 1, 2000, and December 31, 2015, in the National Cancer Center, Korea. Data analysis was performed from December 1, 2019, to May 30, 2020. This study was approved by the institutional review board of the National Cancer Center (No. NCC2020-0140), which waived the need for informed consent because of the retrospective design. This study followed the Strengthening the Reporting of Observational Studies in Epidemiology (STROBE) reporting guideline for cohort studies.

To minimize the stage migration phenomenon and define the criteria for PG, we calculated the incidence of metastasis independently for each LN station, without any reference to the overall pathologic stage.^20^ Predefined criteria for PG that we used to decide the eligibility of PG in this study were (1) location of the tumor in the upper third of the stomach, (2) carcinoma other than Borrmann type 4, and (3) incidence of LN metastasis to key stations was low enough to ignore the need for TG or not lead to expected benefit when dissecting these stations. An incidence of metastasis at key LN stations of more than 1.0% was considered sufficient to indicate dissection.^24^

### Patients

The clinical records of 9952 patients who underwent surgery for gastric carcinoma between December 1, 2000, and December 31, 2015, in the National Cancer Center were evaluated from a prospectively maintained database. Among these, 2347 patients underwent TG for gastric carcinoma and 920 underwent TG for the lesions only in upper third of the stomach.

The inclusion criteria were age between 18 and 85 years, histologically proven adenocarcinoma located in the upper third of the stomach (high body, fundus, or cardia), curative R0 TG performed, and postoperative follow-up for at least 3 years. We excluded patients with a history of other carcinomas (n = 54), combined resection of other organs (n = 54), use of neoadjuvant chemotherapy (NAC) (n = 15), identification of peritoneal seeding at the time of the operation (n = 4), diffuse-type gastric carcinoma (n = 9), clinical T4 category (n = 126), and missing clinical data (n = 3). A total of 655 patients who had gastric adenocarcinomas located in the upper third of the stomach were included in the first statistical analysis.

### Clinical and Pathological Factors

For the staging, the eighth edition TNM classification for gastric carcinoma of the American Joint Committee on Cancer was used.^25^ However, T1a and T1b were grouped and presented as the T1 category. The following clinical and pathologic factors were reviewed: age, sex, location within the upper third of the stomach, longest tumor diameter, histologic type, Borrmann type, ulcer (yes/no), the extent of LN dissection, clinical T category, clinical N category, and presence or absence of metastasis in each dissected LN station. The location was defined according to the Japanese Gastric Cancer Association classification and based on preoperative esophagogastroduodenoscopy reports.^26^ The location within the upper third of the stomach was divided into cardia, fundus, and high body, also according to preoperative esophagogastroduodenoscopy reports. The clinical T and N categories were based on preoperative abdominopelvic computed tomography reports. An ulcer was defined as the presence of type 0 to III for early gastric carcinoma or type 2 and type 3 for advanced gastric carcinoma. The definition of LN stations and dissection was done according to the Japanese Gastric Cancer Association guideline, 5th edition.^3^ The key LN stations (4d, 5, and 6) are those that the surgeon will not dissect when performing D1+ dissection during the PG operation.

### Statistical Analysis

Associations between each clinicopathologic factor and the incidence of LN metastasis for each LN station were analyzed by the Pearson χ^2^ and Mantel-Haenszel tests. The statistical analysis was done in 2 steps: analysis of the 655 cases followed by analysis of 564 cases after exclusion of the N2 and N3 clinically categorized carcinomas. Univariate and multivariate analyses were used for identifying the significant clinical risk factors that might affect the incidence of LN metastasis. Three-year disease-free survival was calculated by the Kaplan-Meier method, and the association with the TNM category was tested by the log-rank test. The LN metastasis rates were calculated by dividing the number of patients with metastasis at that station by the number of patients in whom that station was dissected and with detailed pathologic reports. Findings were considered significant at *P* < .05, with 2-sided testing. Statistical analyses were carried out using SPSS, version 22 (SPSS Inc).

## Results

### First and Second Analyses 

Six-hundred fifty-five patients (462 men [70.5%] and 193 women [29.5%]) were included in the first statistical analysis; mean (SD) age was 57.7 (11.9) years and mean body mass index was 23.81 (3.09) (calculated as weight in kilograms divided by height in meters squared). Table 1 reports the background characteristics and preoperative clinical findings of the study.

**Table 1.** Background Characteristics and Preoperative Clinical Findings of the 655 Patients Included in the First Statistical Analysis

Characteristic	All patients, No. (%)
No.	655
Age, mean (SD), y	57.74 (11.91)
Sex	
Male	462 (70.5)
Female	193 (29.5)
Extent of LN dissection	
D1+	46 (7.0)
D2	153 (23.4)
D2+	456 (69.6)
Tumor location	
Cardia or GEJ	206 (31.5)
Fundus	12 (1.8)
High body	437 (66.7)
Location within wall	
Anterior	69 (10.5)
Posterior	241 (36.8)
Lesser curvature	220 (33.6)
Greater curvature	116 (17.7)
Encircling	9 (1.4)
Borrmann type	
0	440 (67.2)
1	19 (2.9)
2	34 (5.2)
3	162 (24.7)
Differentiation	
Well	123 (18.8)
Moderate	160 (24.4)
Poor and/or SRC	372 (56.8)
Clinical T category	
T1a	190 (29)
T1b	200 (30.5)
T2	105 (16.0)
T3	160 (24.4)
Clinical N category	
N0	436 (66.6)
N1	128 (19.5)
N2	50 (7.6)
N3	41 (6.3)
Size, cm	
≤2	248 (37.9)
2.1-4.0	315 (48.1)
≥4.1	92 (14.0)
Ulcer presence	
Yes	268 (40.9)
No	387 (59.1)
Elevated vs flat	
Elevated	74 (11.3)
Flat	581 (88.7)
ASA score	
1	243 (37.1)
2	379 (57.9)
3	33 (5.0)

Abbreviations: ASA, American Society of Anesthesiologists; GEJ, gastroesophageal junction; LN, lymph node; SRC, signet ring cell carcinoma.

The incidence of LN metastasis in the cN2 and cN3 categories was 5 times more than in the cN0 and cN1 categories, and the hazard ratio (HR) was 5.5 (95% CI, 3.4-8.7; *P* ≤ .001). The overall rate of LN metastasis for the cN2/3 category was 61.5% (56 of 91), and the incidence of metastasis at the key 4d station in the cN2/3 category was 5.6% (4 of 71). This incidence was higher than the cutoff value of the predefined criteria for PG in this study.

Other significant factors were tumor size greater than or equal to 4.1 cm (HR, 4.8; 95% CI, 2.8-8.2; *P* ≤ .001), moderately differentiated carcinoma (HR, 2.8; 95% CI, 1.5-5), poorly differentiated carcinoma (HR, 2.7; 95% CI, 1.3-3.8), T category (T2: HR, 5.3; 95% CI, 3.2-8.8 and T3: HR, 13; 95% CI, 8.78-21.7), and the presence of an ulcer (HR, 3.9; 95% CI, 2.7-5.6).

After the exclusion of patients with N2 and N3 category carcinomas, 564 cases were included in the second statistical analysis (mean [SD] age, 57.8 [11.78] years). The incidences of LN metastasis for these 564 patients are presented in Table 2.

**Table 2.** Incidence of LN Metastasis According to Clinical Stages for 564 Cases

LN station	No./No. (%)							*P* value
	Total	T1N0	T1N1	T2N0	T2N1	T3N0	T3N1	
All nodes	127/564 (22.5)	39/337 (11.6)	2/47 (4.3)	22/61 (36.1)	14/31 (45.2)	20/38 (52.6)	30/50 (60.0)	<.001
1	40/481 (8.3)	10/294 (3.4)	0/38	7/51 (13.7)	2/21 (9.5)	8/36 (22.2)	13/41 (31.7)	<.001
2	22/425 (5.2)	3/255 (1.2)	1/36 (2.8)	3/45 (6.7)	3/18 (16.7)	5/36 (13.9)	7/35 (20.0)	<.001
3	41/443 (9.3)	14/274 (5.1)	0/36	6/44 (13.6)	5/18 (27.8)	7/34 (20.6)	9/37 (24.3)	<.001
4sa	5/388 (1.3)	2/240 (0.8)	0/36	0/42	0/12	3/27 (11.1)	0/31	.07
4b	3/436 (0.7)	0/268	0/37	0/43	0/15	2/35 (5.7)	1/38 (2.6)	.001
4d	3/428 (0.7)	1/264 (0.4)	0/37	0/42	0/17	2/32 (6.3)	0/36	.11
5	0/486	0/295	0/38	0/50	0/22	0/37	0/44	NR
6	2/494 (0.4)	0/300	0/40	1/51 (2.0)	1/21 (4.8)	0/37	0/45	.28
7	27/484 (5.6)	7/294 (2.4)	1/39 (2.6)	2/51 (3.9)	4/21 (19.0)	6/36 (16.7)	7/43 (16.3)	<.001
8a	5/492 (1)	0/296	0/41	2/52 (3.8)	1/20 (5)	1/37 (2.7)	1/46 (2.2)	.009
9	10/464 (2.2)	1/281 (0.4)	0/38	1/46 (2.2)	1/20 (5)	5/36 (13.9)	2/43 (4.7)	<.001
10	1/211 (0.5)	0/106	0/19	0/26	0/13	1/23 (4.3)	0/24	.18
11p	8/418 (1.9)	4/258 (1.6)	0/34	2/44 (4.5)	0/17	2/30 (6.7)	0/35	.52
11d	5/271 (1.8)	0/151	0/27	0/35	0/9	2/22 (9.1)	3/27 (11.1)	<.001
12a	3/356 (0.8)	1/202 (0.5)	0/34	1/38 (2.6)	0/15	0/30	1/37 (2.7)	.31

Abbreviations: LN, lymph node; NR, not relevant.

### Key LN Stations

The rates of LN metastasis of cT1-T3N0/1M0 category tumors for station 5 were 0, irrespective of TN category, tumor size, and differentiation. The overall rate of LN metastasis at station 4d was low (0.7% [3/428]) and not significantly different from the cTNM categories (Table 2). Only 1 patient with category cT1N0M0 (0.4% [1/264]) had a positive node in station 4d in the final pathologic report; this case was 6 cm and poorly differentiated. Station 4d was negative for metastasis for all of the checked cT2N0/1M0 tumors. However, the cT3N0M0 tumor at this station had 2 patients with positive nodes (2 of 32 [6.3%]): both were poorly differentiated adenocarcinomas 2.0 and 4.0 cm at the longest diameter.

Overall, 2 of 294 patients (0.4%) patients had LN metastasis at station 6; this rate was not significantly different between the cTNM categories. One patient had a positive station 6 node in a category cT2N0M0 tumor that was poorly differentiated; this tumor was within the T4a category at the time of surgery and at the final pathologic category. Another positive station 6 node in category cT2N1M0 was moderately differentiated with a 5.0-cm tumor; this case was found to be poorly differentiated in the final pathologic report. With the exclusion of these 2 cases, the incidence of metastasis of station 6 was 0.

In total, 3 of 356 patients (0.8%) had a positive node at station 12a. This rate was not significantly different between the cTNM categories; the category in 1 of 202 patients (0.5%) was cT1N0M0; this case was clinically assessed as a well-differentiated 1.2-cm tumor, but final pathologic analysis defined it as mucinous poorly differentiated carcinoma. One of 38 patients (2.6%) with cT2N0M0 carcinoma who had metastasis at station 12a had a 2.0-cm poorly differentiated carcinoma that, at the final pathologic examination, was categorized as pT3N2M0. In addition, LN metastasis was noted at station 12a in 1 of 37 patients (2.7%) with category cT3N1M0 2-cm poorly differentiated carcinoma.

Station 11d had an overall LN metastasis rate of 1.8% (5 of 271), and we detected a significant difference in the rate of LN metastasis at station 11d when comparing among the TNM categories (Table 2). Station 11d was negative for all cT1-T2/N0/1M0 category carcinomas, while the station 11d incidence of LN metastasis was 9.1% (2 of 22) for cT3N0M0 and 11.1% (3 of 27) for cT3N1M0 poorly differentiated adenocarcinomas.

A Kaplan-Meier curve analysis of 3-year disease-free survival data revealed no significant difference between the cTNM categories or differentiation: disease-free survival was 96.9% for cT1N0/1M0, 95.7% for cT2N0/1M0, and 95.5% for cT3N0/1M0 (*P* = .38); and disease-free survival values for differentiation were 95.5% for well-differentiated, 96.4% for moderately differentiated, and 96.5% for poorly differentiated tumors (*P* = .99).

Moreover, the second logistic regression multivariate analysis failed to show any substantial difference between the N0 and N1 categories. However, tumor size larger than 4.0 cm and differentiation, together with cT category, were still significant factors in general (Table 3). The incidence of tumor metastasis for tumors greater or equal to 4.1 cm compared with less than or equal to 4.0 cm was 3 times more (HR, 2.59; 95% CI, 1.48-4.51; *P* = .001 for univariate analysis and HR, 1.97; 95% CI, 1.03-3.76; *P* = .04 for multivariate analysis). The T3 category had greater incidences of LN metastasis in logistic regression analysis (HR, 11; 95% CI, 6.46-18.73; *P* < .001 for univariate analysis and HR, 10.15; 95% CI, 5.57-18.5; *P* < .001 for multivariate analysis).

**Table 3.** Univariate and Multivariate Logistic Regression Analysis of the Clinical Risk Factors of LN Metastasis for 564 Cases

Clinical risk factor	No. of cases (N = 564)	Univariate analysis		Multivariate analysis	
		HR (95% CI)	*P* value	HR (95% CI)	*P* value
Age, y					
≤60	307	1.08 (0.72-1.6)	.71	1.01 (0.63-1.61)	.97
>60	257	1 [Reference]		1 [Reference]	
Sex					
Male	397	0.89 (0.58-1.36)	.60	0.85 (0.51-1.4)	.53
Female	167	1 [Reference]		1 [Reference]	
Tumor size, cm					
≤2.0	236	1 [Reference]	<.001	NR^a^	
2.1-4.0	268	1.75 (1.23-2.75)	.01		
≥4.1	60	3.58 (1.92-6.69)	<.001		
Tumor size, cm					
≤4.0	504	1 [Reference]		1 [Reference]	
≥4.1	60	2.59 (1.48-4.54)	.001	1.97 (1.03-3.76)	.04
Differentiation					
WD	113	1 [Reference]	.01	1 [Reference]	.03
MD	137	2.81 (1.43-5.5)	.003	2.81 (1.33-5.95)	.007
PD/SRC	314	2.18 (1.17-4.0)	.01	2.15 (1.05-4.38)	.04
cT category					
T1	384	1 [Reference]	<.001	1 [Reference]	<.001
T2	92	5.37 (3.17-9.13)	<.001	5.35 (3.06-9.35)	<.001
T3	88	11 (6.46-18.73)	<.001	10.15 (5.57-18.5)	<.001
cN category					
N0 (0)	436	1 [Reference]	<.001	1 [Reference]	.88
N1 (1-2)^b^	128	0.4 (0.26-0.62)	.002	0.95 (0.55-1.64)	
Ulcer					
Yes	375	1 [Reference]	.76	1 [Reference]	
No	189	1.07 (0.7-1.62)		0.93 (0.57-1.54)	.80
Type					
Elevated	69	1 [Reference]	.049	1 [Reference]	
Flat	495	0.6 (0.33-0.99)		0.60 (0.30-1.18)	.14
Location					
Cardia/GEJ	179	1 (0.68-1.59)	.98	1.12 (0.68-1.82)	.90
Fundus	10	0.00	.84	0.00	.65
HB	375	1 [Reference]	>.99	1 [Reference]	>.99
Location within the wall					
Anterior	62	1 [Reference]	.43	NR^a^	
Posterior	213	0.63 (0.32-1.21)	.17		
Lesser curvature	187	0.79 (0.41-1.52)	.48		
Greater curvature	95	0.84 (0.40-1.75)	.65		
Encircling	7	1.98 (0.40-9.8)	.40		

Abbreviations: GEJ, gastroesophageal junction; HB, high body; LN, lymph node; MD, moderately differentiated; NR, not relevant; PD, poorly differentiated (include signet ring cell carcinoma); WD, well differentiated.

^a^ Multivariate analysis was not performed.

^b^ One or 2 lymph nodes.

### Size and Differentiations

Tumor size can affect the choice of surgery if it is larger than the expected size and does not allow the surgeon to save more than half of the stomach. When analyzing the incidences of LN metastasis for the total number of dissected nodes (all LN stations combined) with a focus on tumor size, the overall incidence of LN metastasis was significant in tumors larger than 4.1 cm in the longest diameter (103 of 504 [20.4%] vs 24 of 60 [40.0%], *P* = .001). The incidence of LN metastasis in key stations (4d, 5, and 6) was low in adenocarcinomas less than or equal to 4.0 cm (2 of 385 [0.5%] for station 4D, 0 of 437 for station 5, and 1 of 445 [0.2%] for station 6). In contrast, the incidence of LN metastasis was higher than 1.0% in tumors greater than or equal to 4.1 cm (1 of 43 [2.3%] for station 4d, 0 of 49 for station 5, and 1 of 49 [2.0%] for station 6). The LN metastatic rates were 1.6% (4 of 247) for tumors less than 4.1 cm and 4.2% (1 of 24) for tumors greater than or equal to 4.1 cm in station 11d. For station 12a, this incidence was not significantly different and was low (3 of 356 [0.8%]); all tumors were less than 4.1 cm. Table 4 reports the incidence of LN metastasis according to tumor size and differentiation.

**Table 4.** Incidence of LN Metastasis According to Primary Tumor Size and Differentiation for 564 Cases

LN station	No./No. (%)			*P* value	No./No. (%)			*P* value
	Total (N = 564)	≤4.0 cm^a^	≥4.1 cm		WD	MD	PD	
All nodes	127/564 (22.5)	103/504 (20.4)	24/60 (40.0)	.001	14/113 (12.4)	39/137 (28.5)	74/314 (23.6)	.06
1	40/481 (8.3)	31/431 (7.2)	9/50 (18.0)	.009	4/101 (4.0)	18/114 (15.8)	18/266 (6.8)	.95
2	22/425 (5.2)	14/381 (3.7)	8/44 (18.2)	.001	2/87 (2.3)	9/114 (8.9)	11/237 (4.6)	.74
3	41/443 (9.3)	32/397 (8.1)	9/46 (19.6)	.01	4/94 (4.3)	15/102 (14.7)	22/247 (8.9)	.44
4sa	5/388 (1.3)	4/348 (1.1)	1/40 (2.5)	.47	0/77	2/94 (2.1)	3/217 (1.4)	.50
4b	3/436 (0.7)	3/392 (0.8)	0/44	.56	0/88	0/103	3/245 (1.2)	.16
4d	3/428 (0.7)	2/385 (0.5)	1/43 (2.3)	.18	0/88	0/100	3/240 (1.3)	.16
5	0/489	0/437	0/49	NR	0/101	0/117	0/268	NR
6	2/494 (0.4)	1/445 (0.2)	1/49 (2.0)	.06	0/103	1/121 (0.8)	1/270 (0.4)	.78
7	27/484 (5.6)	24/436 (5.5)	3/48 (6.3)	.83	2/101 (2.0)	6/118 (5.1)	19/265 (7.2)	.05
8a	5/492 (1.0)	3/445 (0.7)	2/47 (4.3)	.02	1/105 (9.5)	2/117 (1.7)	2/270 (0.7)	.71
9	10/464 (2.2)	8/418 (1.9)	2/46 (4.3)	.28	0/94	2/113 (1.8)	8/257 (3.1)	.07
10	1/211 (0.5)	1/184 (0.5)	0/27	.70	0/38	0/57	1/116 (0.8)	.41
11p	8/418 (1.9)	5/376 (1.3)	3/42 (7.1)	.009	0/82	2/105 (1.9)	6/231 (2.6)	.16
11d	5/271 (1.8)	4/247 (1.6)	1/24 (4.2)	.38	0/55	0/63)	5/153 (3.3)	.07
12a	3/356 (0.8)	3/322 (0.9)	0/34	.57	1/63 (1.6)	1/93 (1.1)	1/200 (0.5)	.38

Abbreviations: LN, lymph node; MD, moderate differentiation; NR, not relevant; PD, poorly differentiated (include signet ring cell carcinoma); WD, well differentiated.

^a^ A cutoff value of 4.0 cm was used based on logistic regression analysis, and it is difficult to perform limited resection and save a safe margin in tumors larger than 4.1 cm.

The well-differentiated carcinomas had the lowest overall LN metastasis incidence, which appeared in 14 of 113 cases (12.4%) with 0 incidence at the key stations irrespective of cTNM category and tumor size (Table 4). Only 1 patient with moderately differentiated carcinomas (1 of 121 [0.8%]) was observed to have a positive node at all 6 stations. This case was initially classed as a 5-cm, category cT2N1M0 moderately differentiated carcinoma, but the final pathologic testing revealed poorly differentiated carcinoma. Stations 4d and 5 had 0 metastases in the moderately differentiated tumors, irrespective of cTNM category or size.

Poorly differentiated carcinoma was a risk factor for metastasis in station 4d for 3 of 240 patients (1.3%) (1 patient with cT1N0M0 6-cm carcinoma, and 2 patients (0.8%) with cT3N0M0 carcinoma, both less than 4.1 cm in diameter). Station 6 was positive in 1 of 270 patients (0.4%) with a 1.5-cm category cT2N0M0 poorly differentiated tumor. Table 4 presents the details of LN metastasis incidence according to differentiation.

We observed that the incidence of metastasis at key stations was less than 1.0%, which suggests that PG with D1 dissection can be safely performed in clinically differentiated category T1N0/1M0 upper third gastric carcinoma tumors smaller than 4.1 cm. Proximal gastrectomy with D1+ dissection can be safely performed in poorly differentiated category cT1N0/1M0, cT2N0/1M0, and differentiated cT3N0/1M0 carcinomas of the upper third of the stomach.

For poorly differentiated category cT3N0/1M0 tumors, because stations 4d, 11d, and 12a need to be dissected, in addition to the D1+, it seems that TG is a more appropriate procedure than PG. The Figure proposes a selection diagram for PG eligibility.

**Figure.**
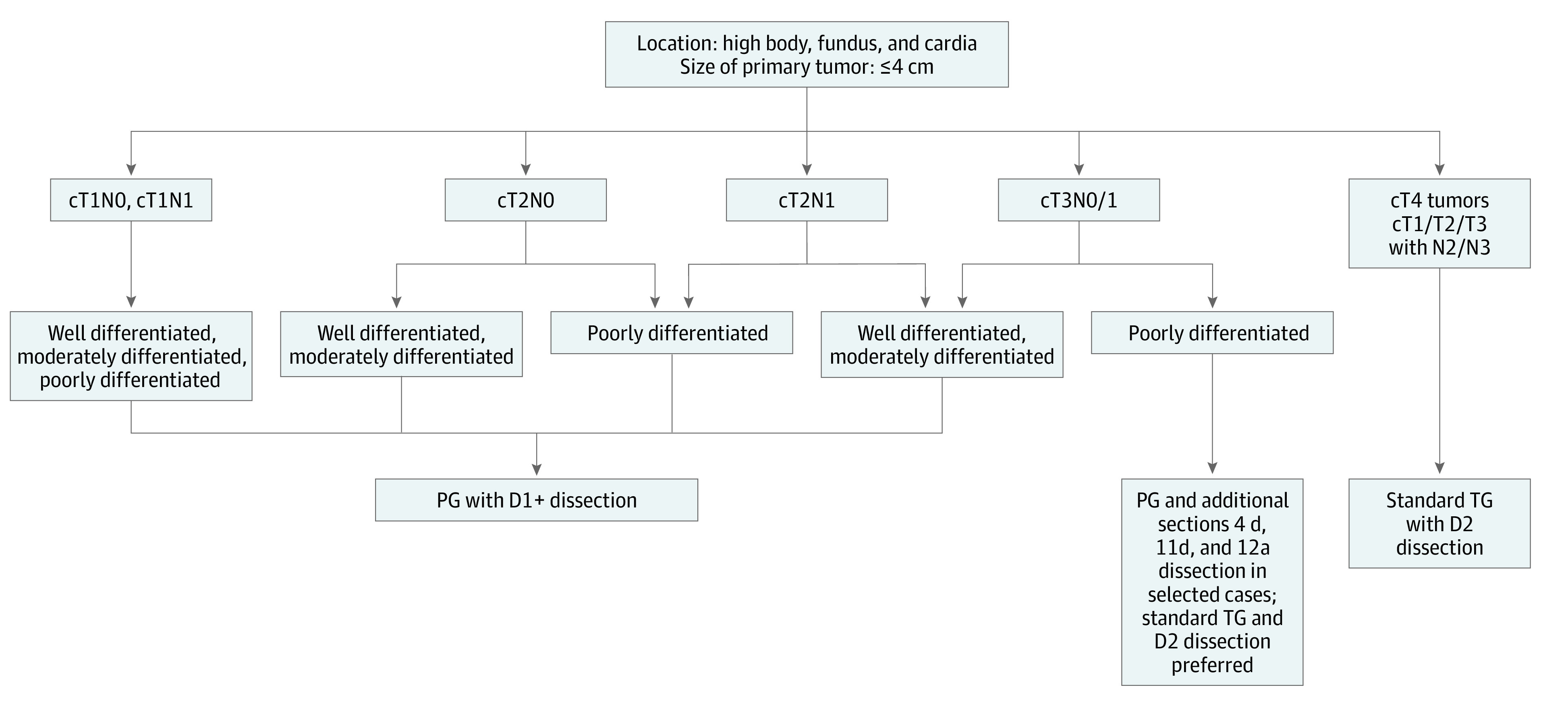
Treatment Algorithm for Upper Third Gastric Carcinoma Tumors Less Than or Equal to 4.0 cm D1 dissection: Nos. 1, 2, 3, 4sa, 4sb, & 7; D1+ dissection: Nos. 1, 2, 3, 4sa, 4sb, 7, 8a, 9, & 11p as described in the Japanese Gastric Cancer Association treatment guideline, 5th edition.^3^ PG indicates proximal gastrectomy; TG, total gastrectomy.

## Discussion

Proximal gastrectomy saves physiologic function and improves the patient’s quality of life, nutritional status, weight maintenance, morbidity, and anemia compared with TG.^5,6,7,8,9,10,11,12,13,14,15^ In this study we attempted to develop an indication for the PG operation based on the risk for LN metastasis to key LN stations. We found that the rates of LN metastasis at key stations for upper third gastric carcinoma are negligible, which suggest that the PG with D1 dissection can be safely performed in clinically differentiated category T1N0/1M0 upper third adenocarcinomas less than 4.1 cm. Proximal gastrectomy with D1+ dissection could be safely performed in poorly differentiated cT1N0/1M0, cT2N01M0, and differentiated cT3N0/1M0 carcinomas of the upper third of the stomach when the tumor is less than 4.1 cm in diameter. For the poorly differentiated category cT3N0/1M0 tumor, there is a risk of metastasis at stations 4d and 11d and lower risk at station 12a. Thus, PG with D1+ dissection and additional dissection of stations 4d, 11d, and 12a would be considered a possible procedure for carcinomas less than 4.1 cm. However, because of the location of the 4d station along the middle and lower body of the stomach, TG is recommended.

Only 2 cases had tumor metastasis at station 6: 1 understaged preoperatively as T2 rather than T4a, and the second poorly differentiated tumor was larger than 5 cm in diameter. Any surgeon encountering this surgical stage migration during the operation could change the procedure to a more radical TG.

Regarding station 4d, the rate of LN metastasis for the T3 category was 6.3%, and both poorly differentiated tumors were located close to the greater curvature in the high body of the stomach. The rate of LN metastasis at station 4d was not statistically significantly different when compared among cTNM categories. The location in the greater curvature of the stomach could explain this lack of significance because of the flow of the lymphatic drainage. This station should be dissected for T3 poorly differentiated carcinomas, especially when tumor location is in the greater curvature. Similarly, we recorded a low rate of LN metastasis (0.8%) for station 12a, and for cT3N1M0 tumors—this rate did not exceed 2.7% (Table 2). Also, it is straightforward to dissect station 12a when considering PG for advanced cT2-T3N0/1M0 poorly differentiated carcinoma of the upper third of the stomach.

A significant increase in the rates of LN metastasis (up to 9.1%-11.1%) was observed in category cT3N0/1M0 tumors at station 11d. Thus, it seems that the dissection of this group in category cT3 tumors is necessary. However, dissection of station 11d is possible and straightforward when performing PG. Therefore, we believe that the increased likelihood of LN metastasis at station 11d should not be a contraindication to PG in category cT3N0/1M0 tumors, but rather should be an indication for PG with the need to dissect that station. A similar rate for station 11d from category cT3 upper third gastric carcinoma has been reported.^17^

We observed that a tumor size greater than 4.1 cm is a risk factor for metastasis (Table 4). Moreover, the tumor size is important because, if the tumor is too extended, it will be difficult to achieve a free margin while also saving important physiologic functions of the remnant stomach and achieving functional benefits over TG. The rates of metastasis to the key stations, as well as station 12a, are negligible and close to 0 for tumors smaller than 4.1 cm, and low (1.6%) even for station 11d.

Our results support other reports.^17,18,19,20,21,22,23^ Yura et al^17^ suggested that PG is safe in the T2-T3 pathologic category and noted a similar rate of LN metastasis in the evaluation of 202 TG cases. Likewise, in pathologically categorized T1-T4 upper third gastric carcinoma, Haruta et al^18^ found low metastatic rates at the key stations plus station 12a. This rate was 0 even for T1-T2 carcinomas and, for T3 carcinomas, 3.3% for station 4d, 0.5% for station 5, and 1.6% for station 6. Similarly, these authors included N2 and N3 carcinomas, and it is not clear whether these low rates reflect carcinomas of N0-N1 rather than N2-N3 categories, which we excluded. Sugoor et al^19^ examined the oncologic safety of PG for pathologically T3 category tumors by evaluating the recurrence rate and overall survival and, unlike in our study, included patients after neoadjuvant chemotherapy.

Sasako et al^20^ reported low therapeutic indices for stations 5 and 6 of upper third gastric carcinoma. Ooki et al^21^ presented similar results of LN metastasis to individual stations. At category T2, the rate of metastasis in 27 patients at key stations was 0. The authors noted that category T3 upper third gastric carcinoma (n = 82) had low metastatic rates at stations 4d (3.7%), 5 (2.4%), and 6 (0%), but suggested that PG is eligible in category T2 tumors. Also, similar to our findings, these authors reported a rate of 9.8% at station 11 for subserosal carcinoma (category T3) but did not distinguish station 11p from 11d. This dissimilar rate likely accounted for the different conclusions reached by us and Ooki et al. We believe station 11d is not a key station and can be dissected during PG for patients with category T3 carcinoma, avoiding the more morbid TG operation for patients with tumors smaller than 4.0 cm.

### Strengths and Limitations

Strengths of this study include its large number of cases that underwent only curative resection; we used the clinical rather than pathologic category because surgeon decisions are clinically based, and some discrepancies between clinical and pathologic staging are unavoidable. Also, every LN station metastasis was reviewed individually, considering the size and differentiation.

This study has limitations. First, it was retrospective, and some selection biases might exist. Second, not all patients had detailed LN data available, and we included cases with large tumors that could not be candidates for PG because of consideration for the volume of the remnant stomach. Despite this limitation, the rate of metastasis to key stations remained negligible.

## Conclusions

We have developed a preliminary selection diagram for PG eligibility (Figure). The findings of this study suggest that PG can be safely performed for cT1-T2N0/1M0 tumors less than 4.1 cm in diameter that are located in the upper stomach. The cT3N0/1M0-diffrentiated tumors less than 4.1 cm were also eligible for PG, whereas poorly differentiated cT3 tumors and any cT4 or cN2/3 disease require TG.
